# Supplementary material for: Synthesis and Biological Activity of Benzamides Substituted with Pyridine-Linked 1,2,4-Oxadiazole
Source: Molecules. 2020 Jul 31;25(15):3500. doi: 10.3390/molecules25153500 (PMC7435590; doi:10.3390/molecules25153500)
Supplement: Supplementary file 1 [file molecules-25-03500-s001.pdf]

Supporting Information

Synthesis and Biological Activity of Benzamides Substituted with Pyridine-linked 1,2,4-oxadiazole

Sen Yang, Xiao-Yu Tian, Tian-Yang Ma, Li Dai, Chao-Li Ren, Jun-Chang Mei, Xing-Hai Liu, Cheng-Xia, Tan*

1. ^{1}H NMR spectra of **7a~7n**……………………………………S1-S14
2. ^{13}C NMR spectra of **7a~7n**………………………………….S15-S28
3. ESI-HRMS spectra of **7a~7n**……………………………..….S29-42

1. ^{1}H NMR spectra of **7a~7n**

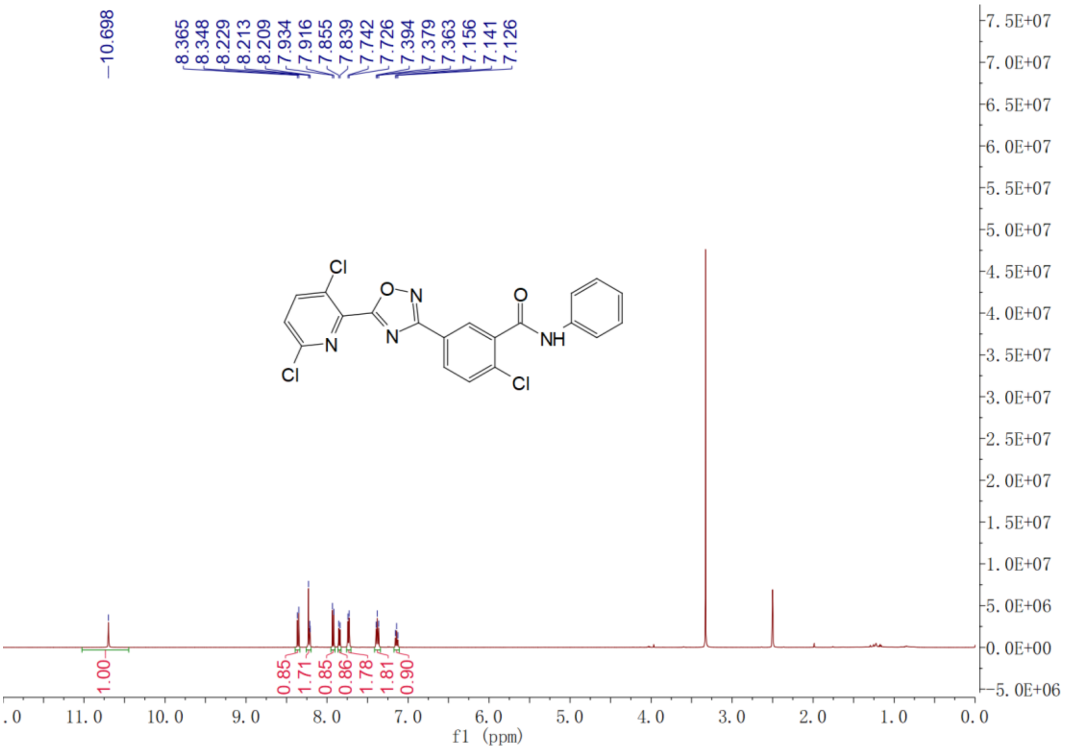

Figure S1 ^{1}H NMR spectra of **7a**

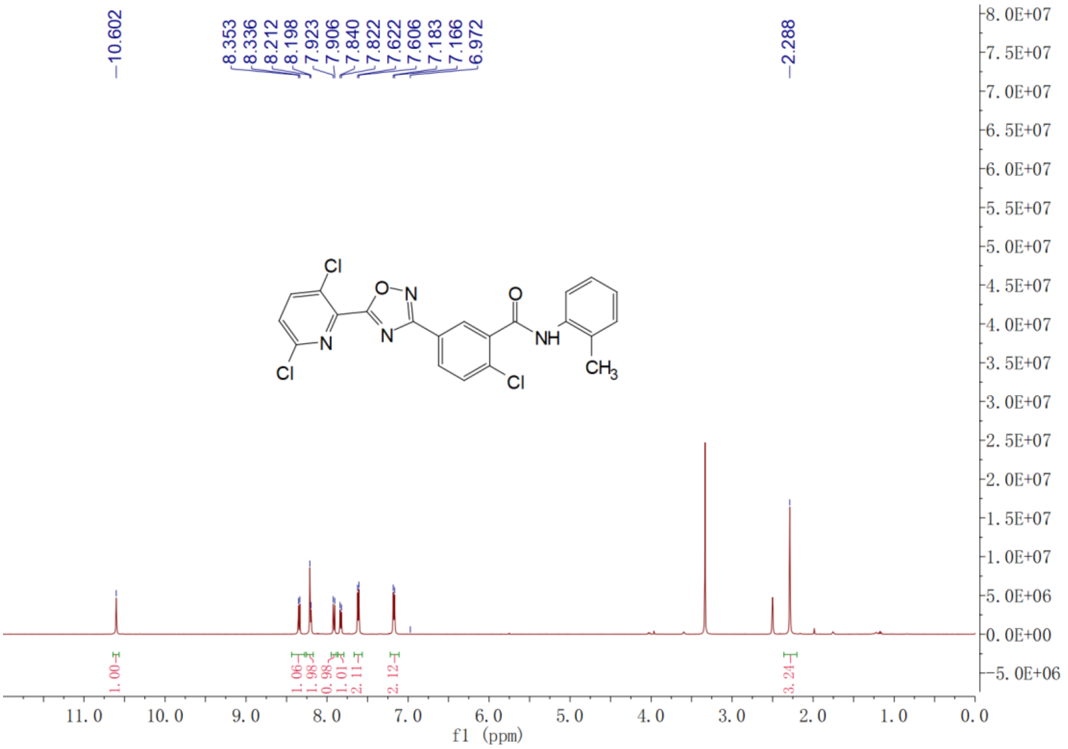

Figure S2 ^{1}H NMR spectra of **7b**

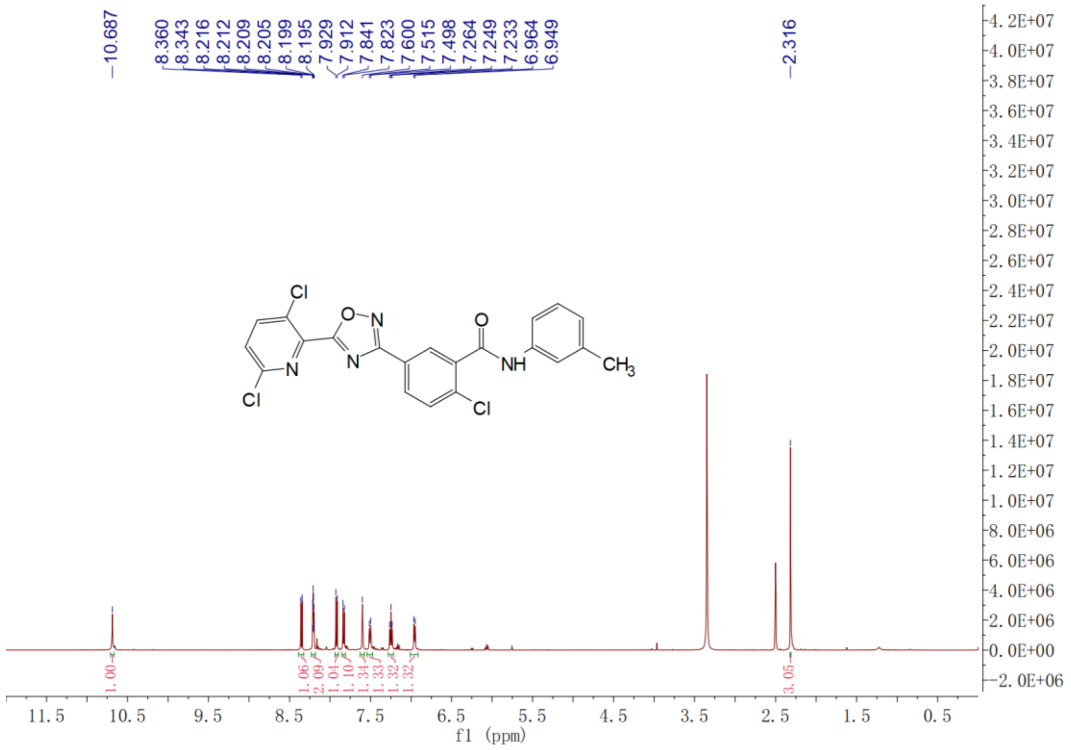

Figure S3 ^{1}H NMR spectra of **7c**

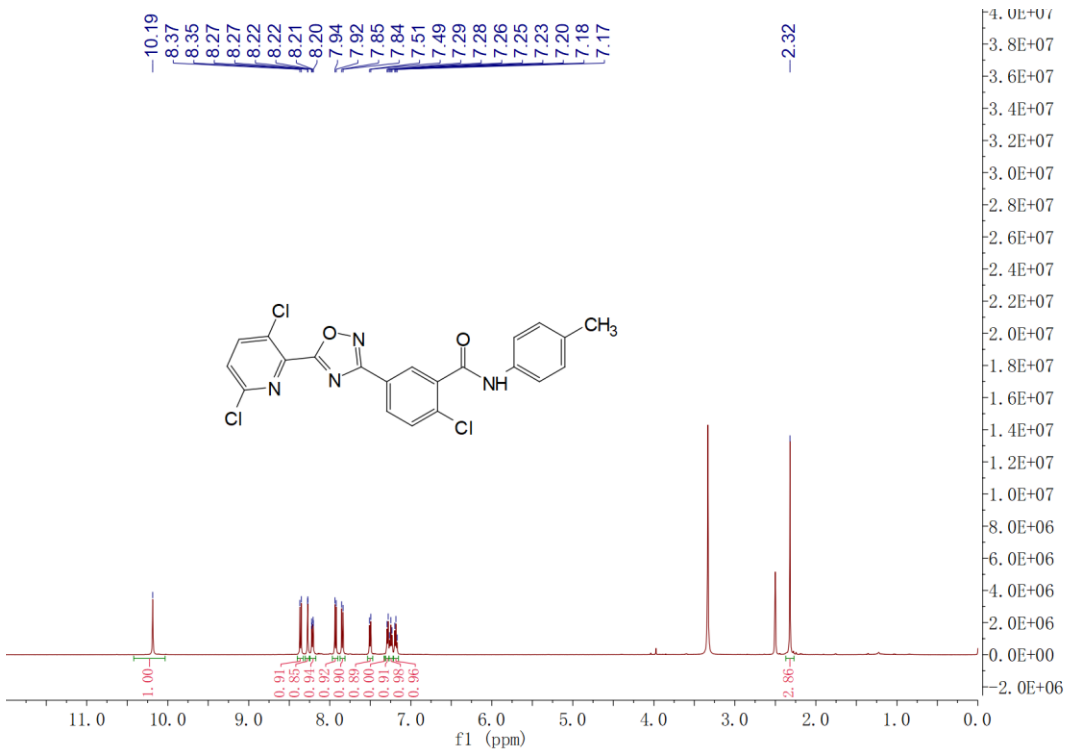

Figure S4 ^{1}H NMR spectra of **7d**

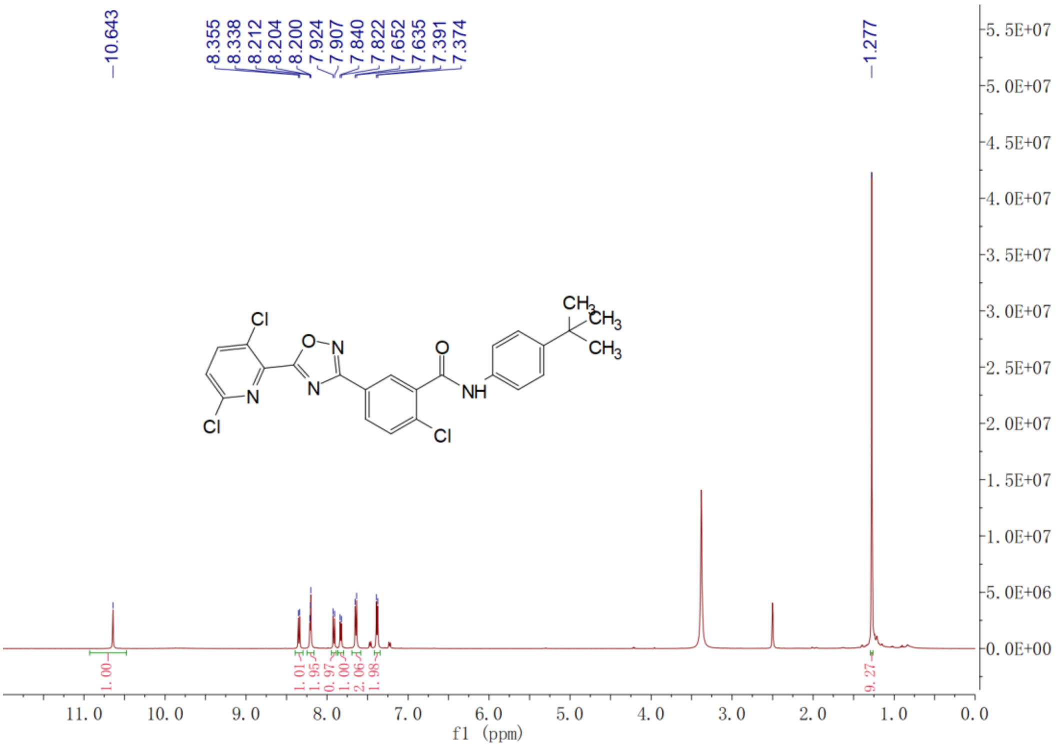

Figure S5 ^{1}H NMR spectra of **7e**

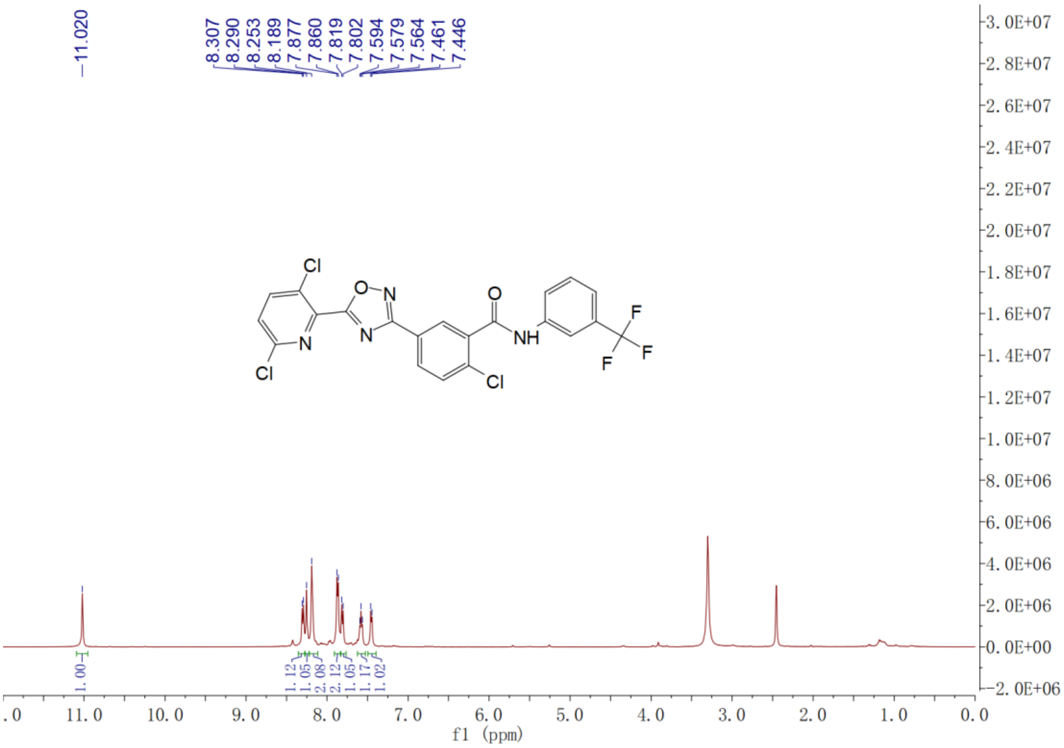

Figure S6 ^{1}H NMR spectra of **7f**

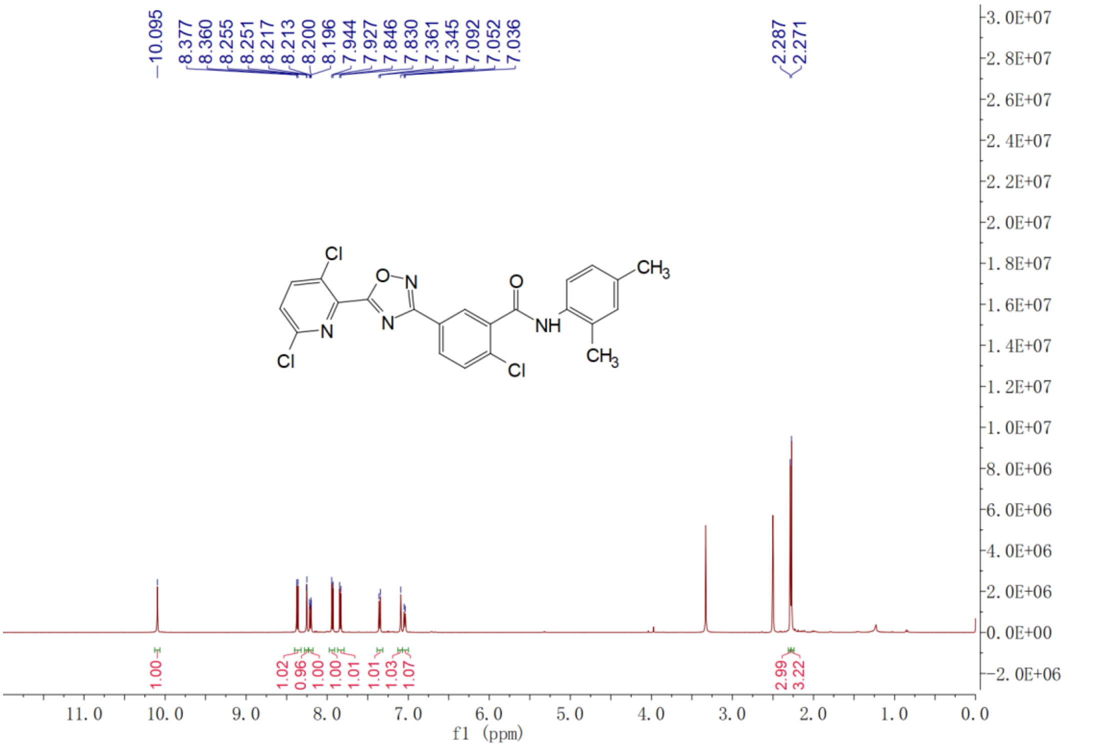

Figure S7 ^{1}H NMR spectra of **7g**

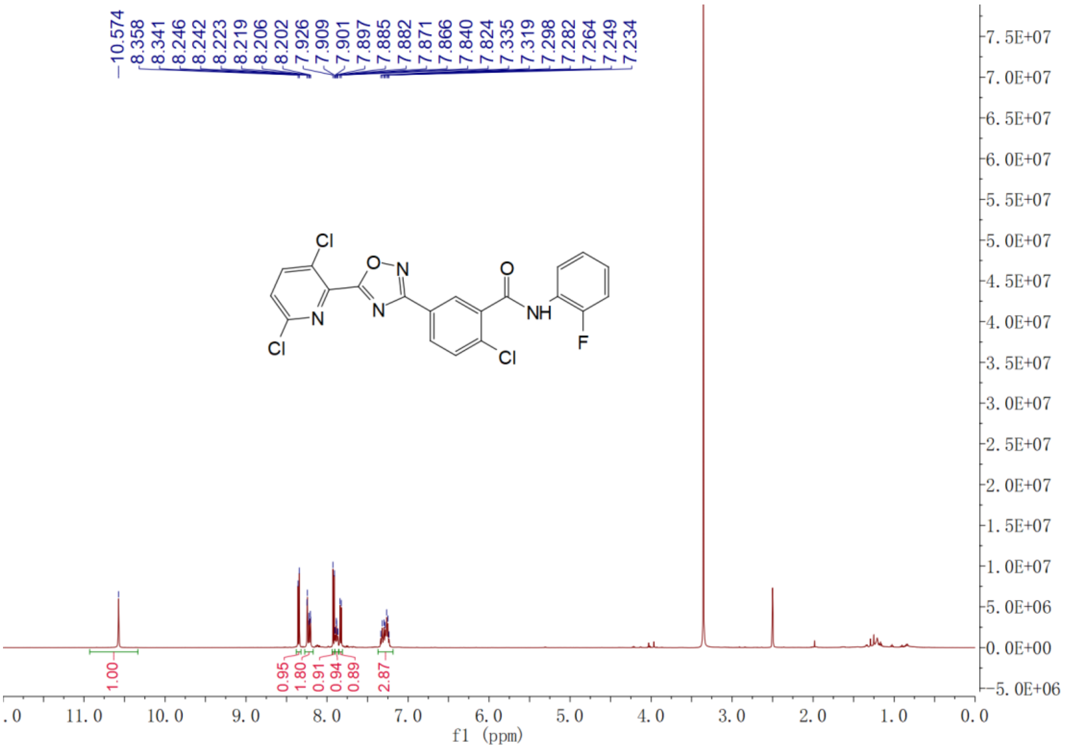

Figure S8 ^{1}H NMR spectra of **7h**

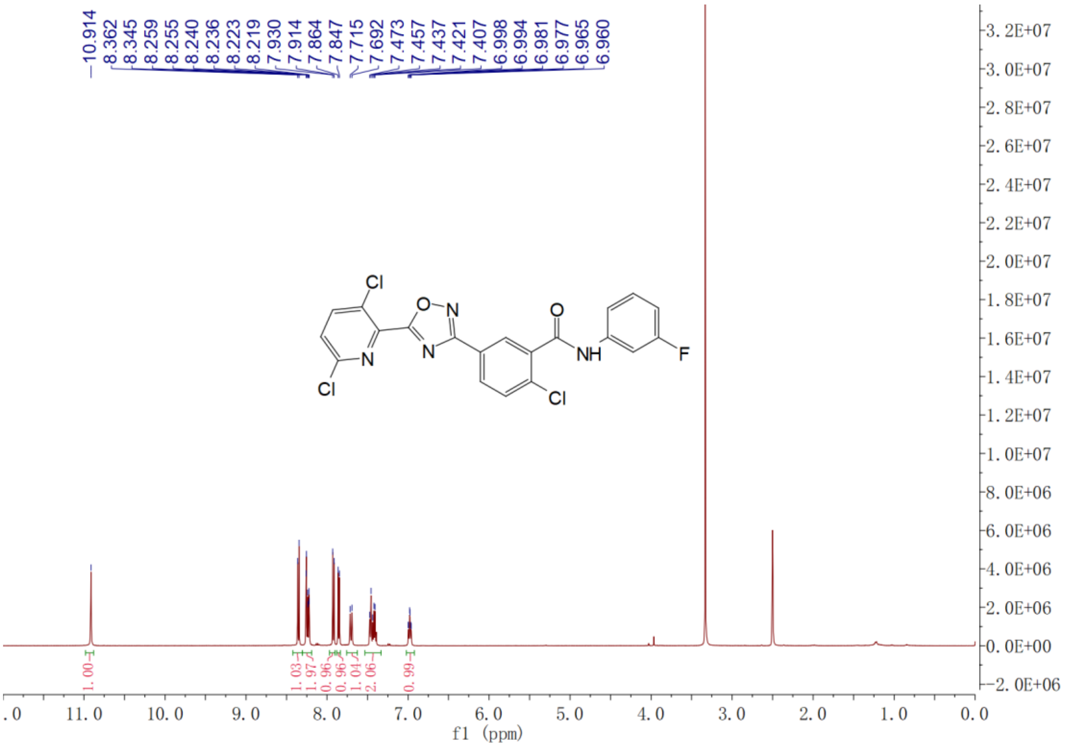

Figure S9 ^{1}H NMR spectra of **7i**

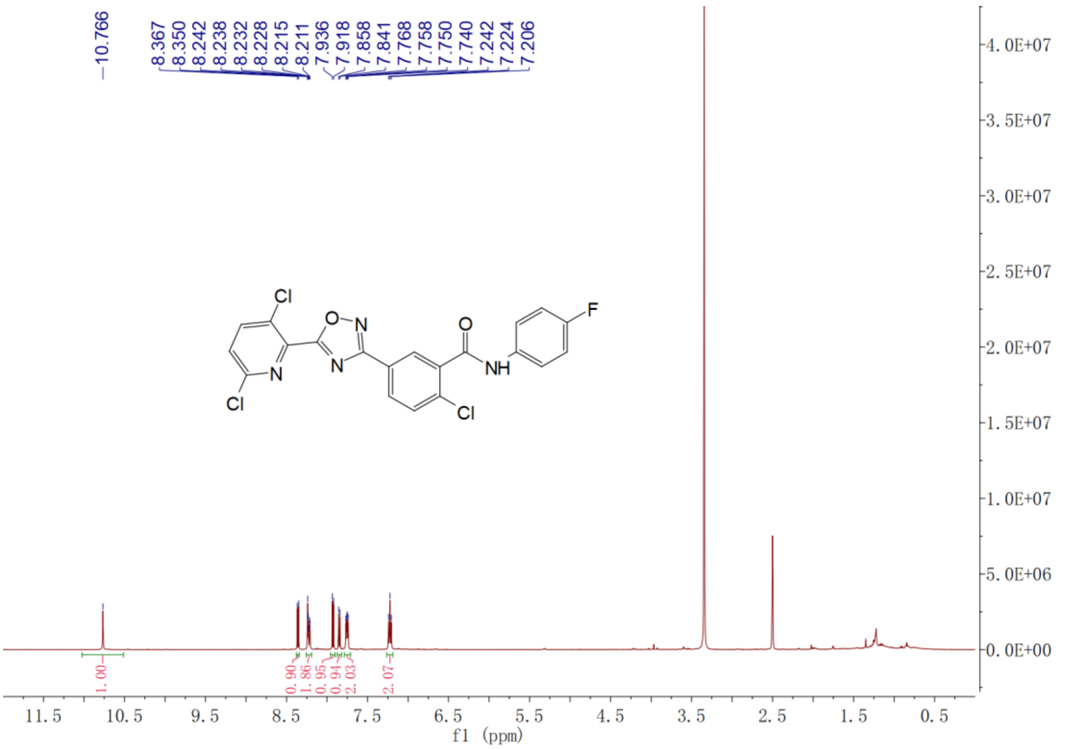

Figure S10 ^{1}H NMR spectra of **7j**

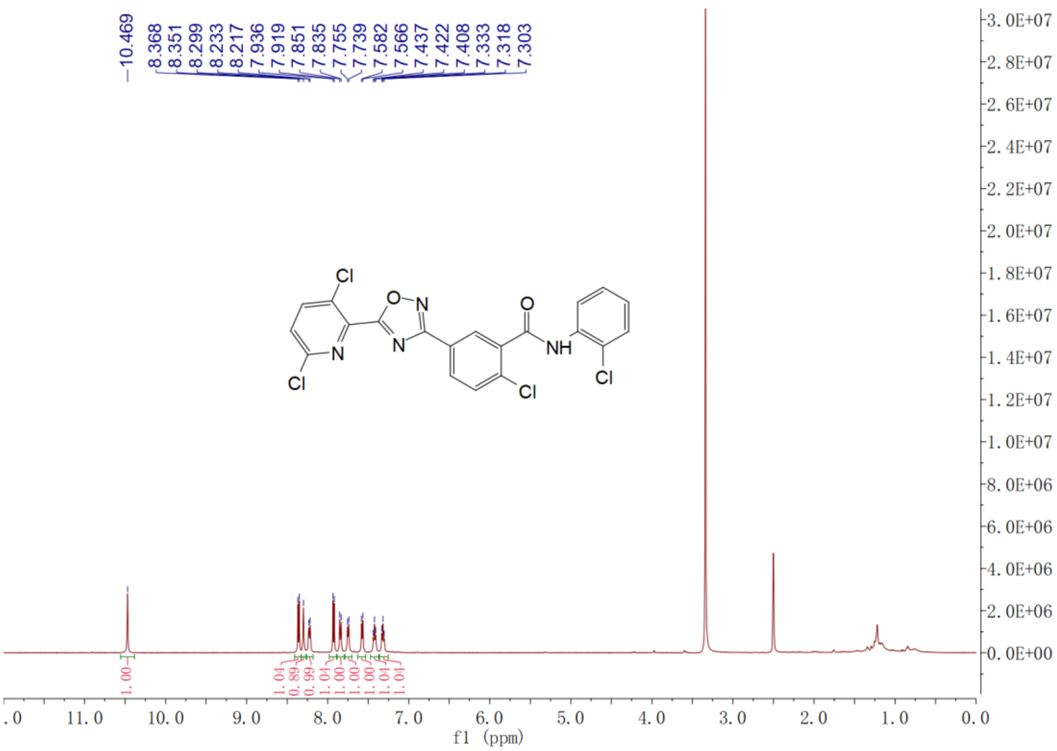

Figure S11 ^{1}H NMR spectra of **7k**

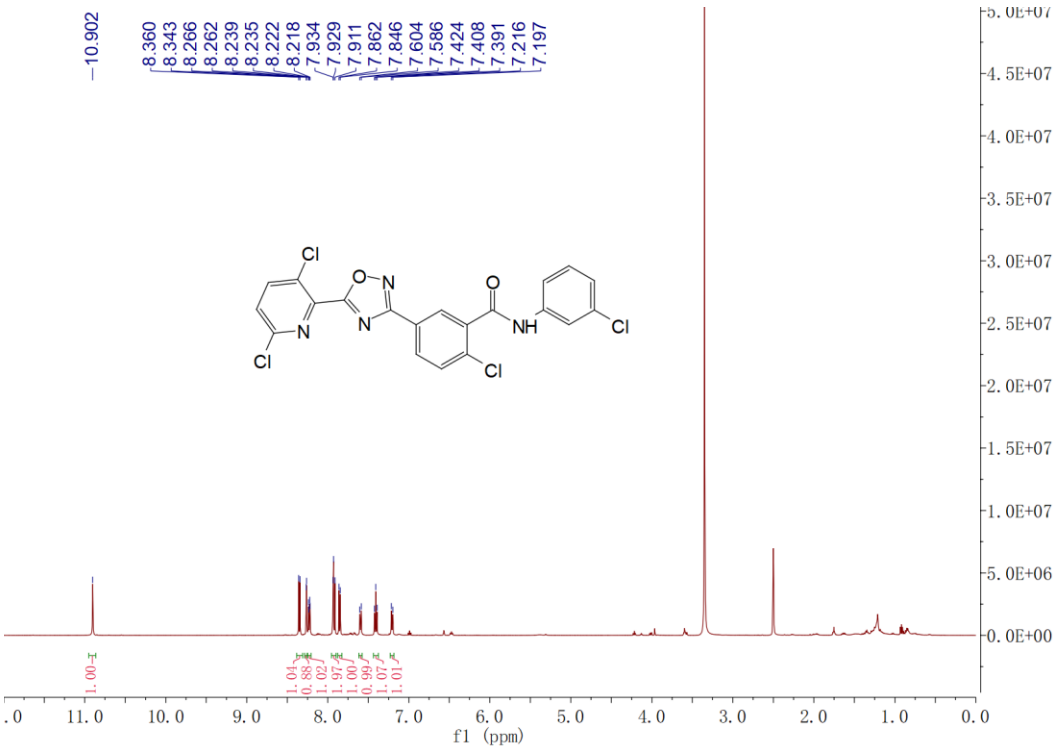

Figure S12 ^{1}H NMR spectra of **7l**

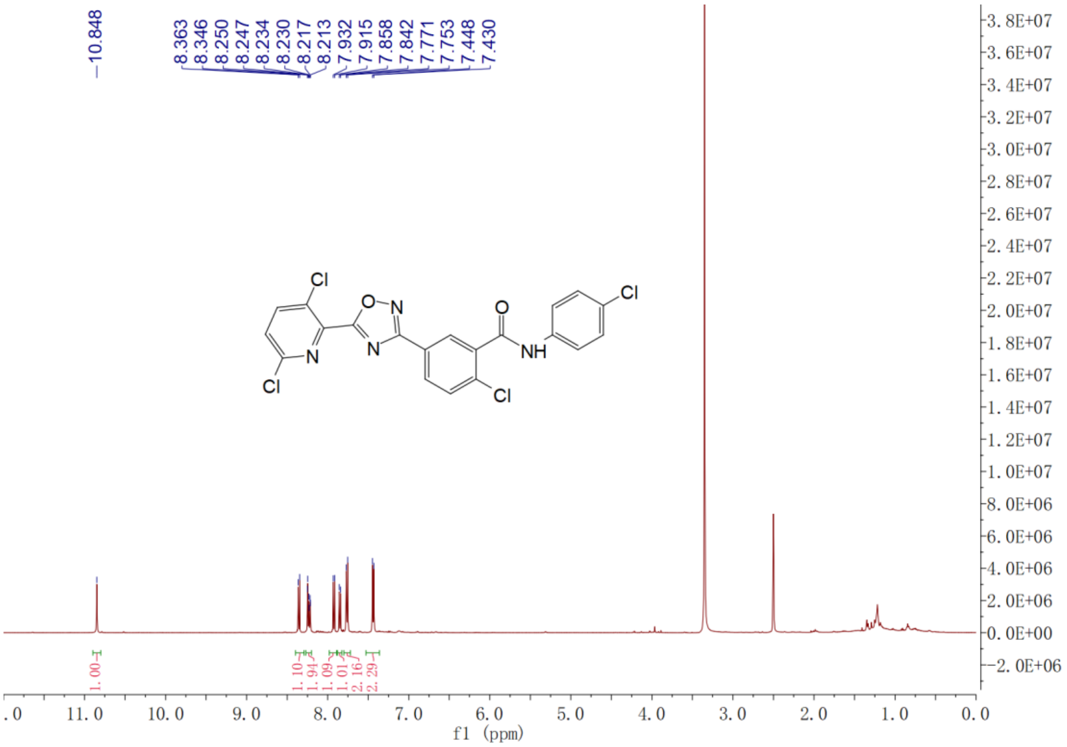

Figure S13 ^{1}H NMR spectra of **7m**

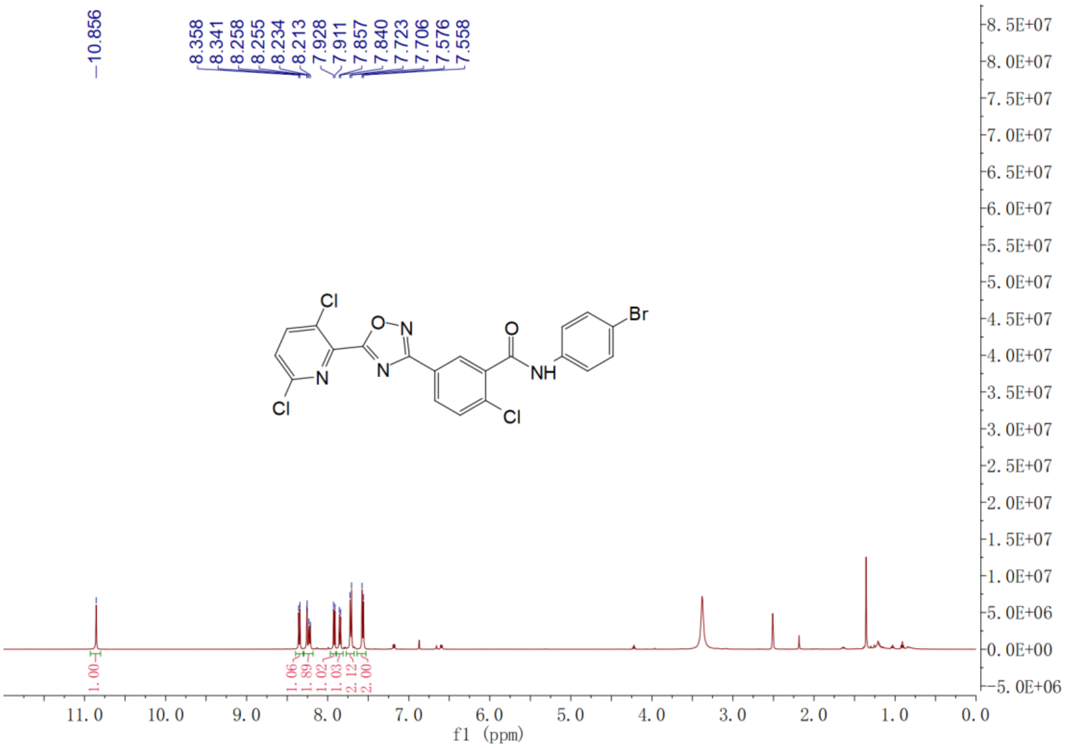

Figure S14 ^{1}H NMR spectra of **7n**

2. ^{13}C NMR spectra of **7a~7n**

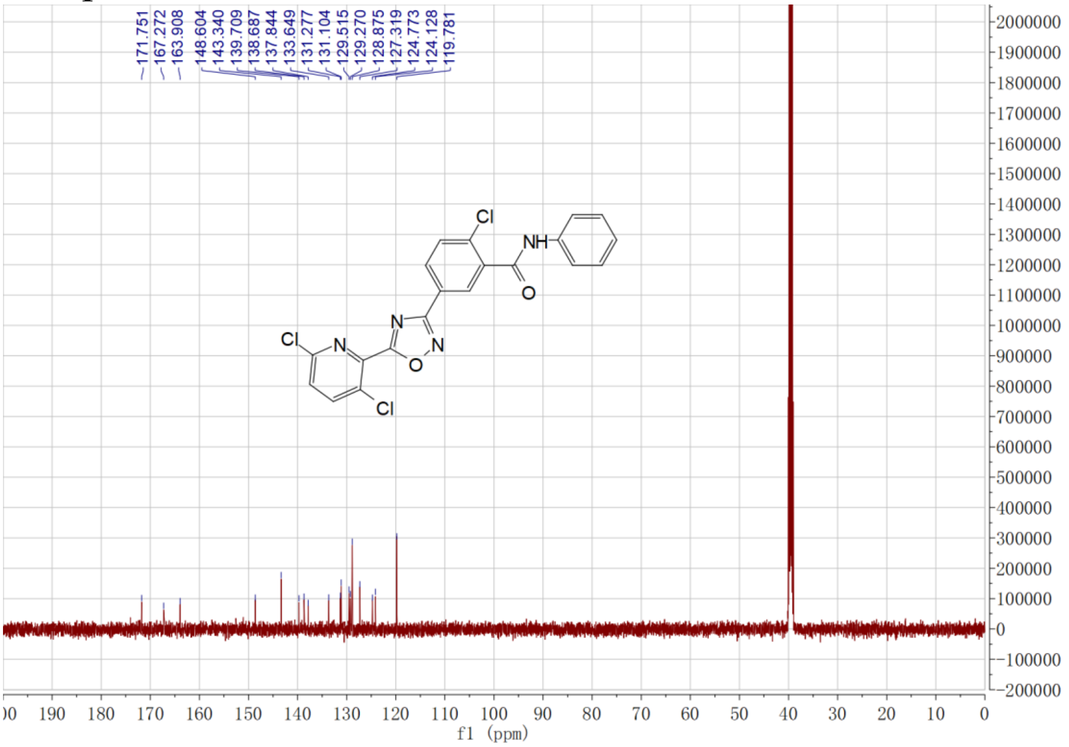

Figure S15 ^{13}C NMR spectra of **7a**

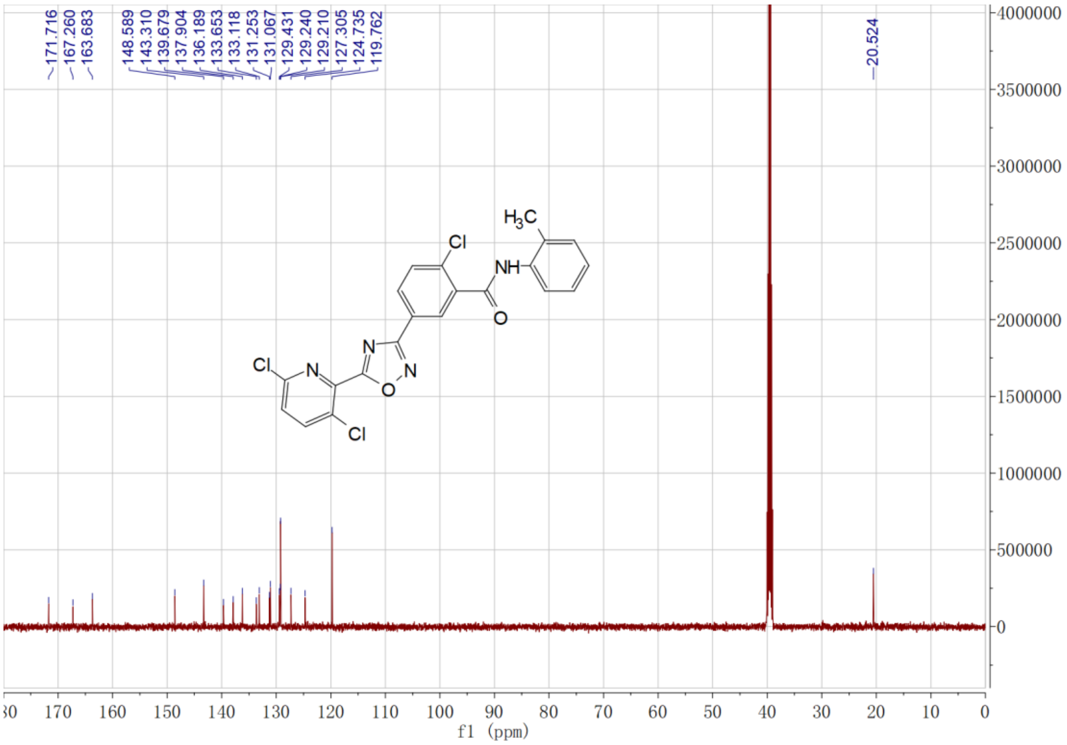

Figure S16 ^{13}C NMR spectra of **7b**

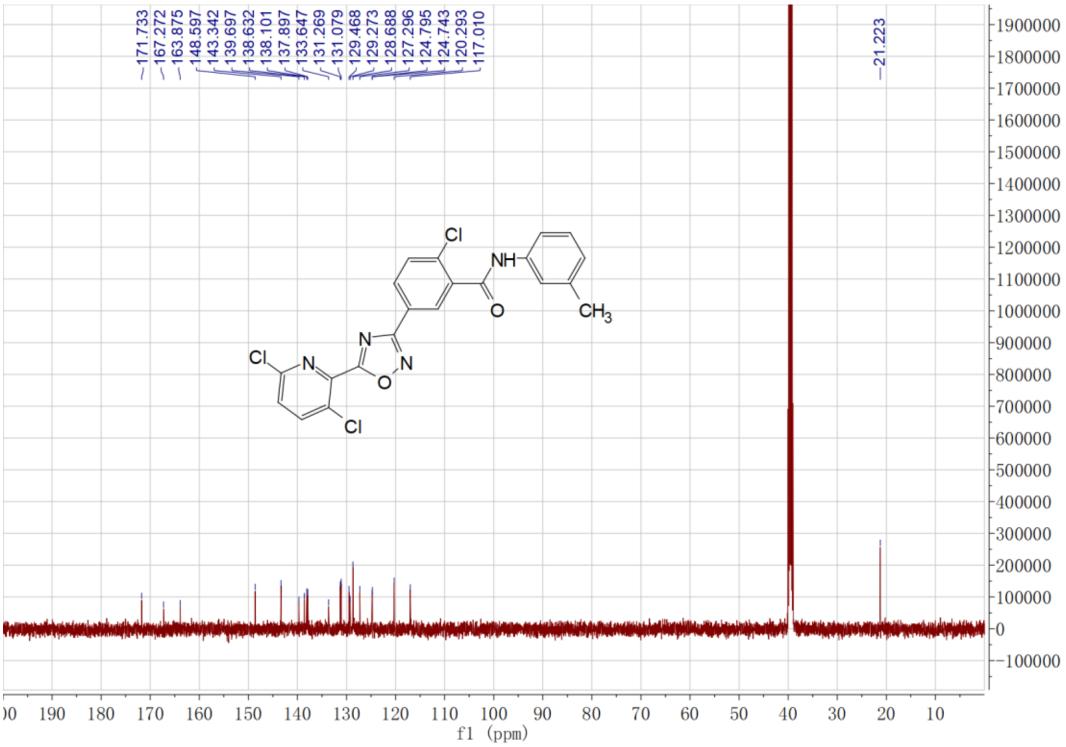

Figure S17 ^{13}C NMR spectra of **7c**

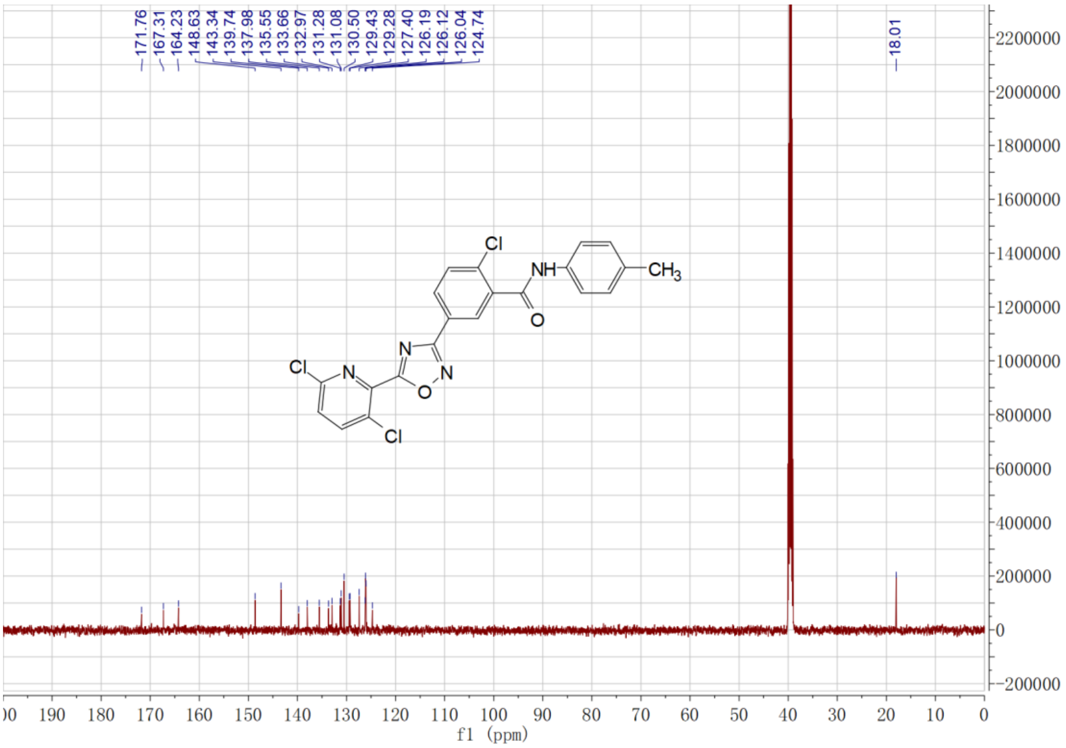

Figure S18 ^{13}C NMR spectra of **7d**

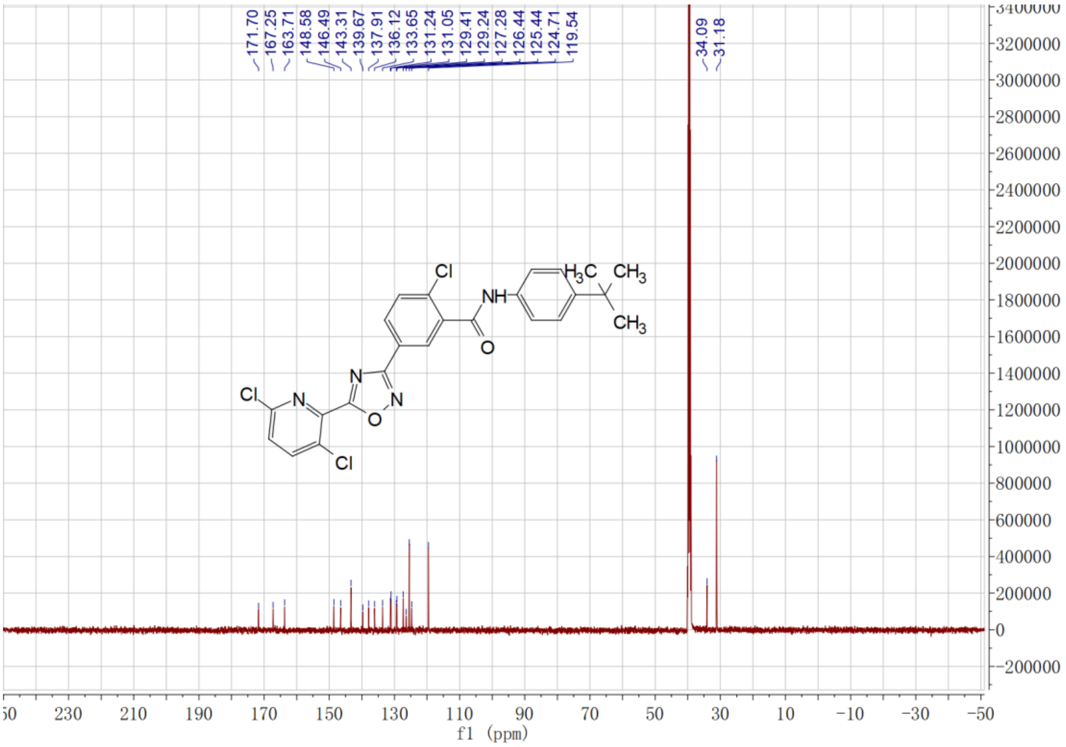

Figure S19 ^{13}C NMR spectra of **7e**

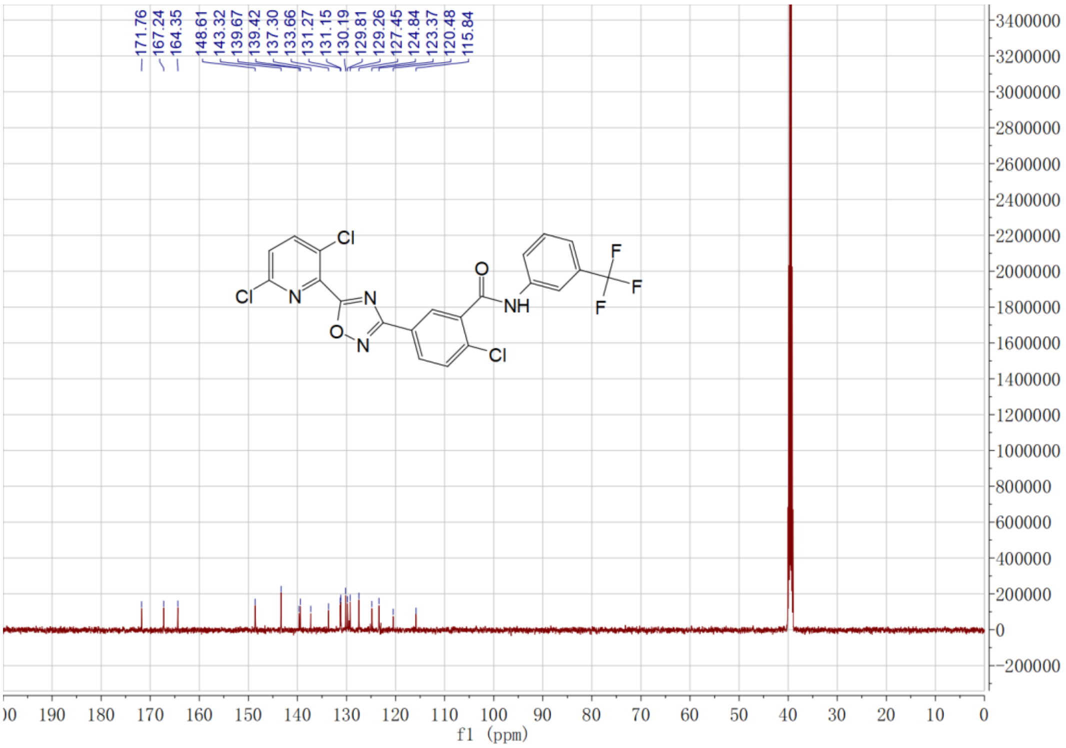

Figure S20 ^{13}C NMR spectra of **7f**

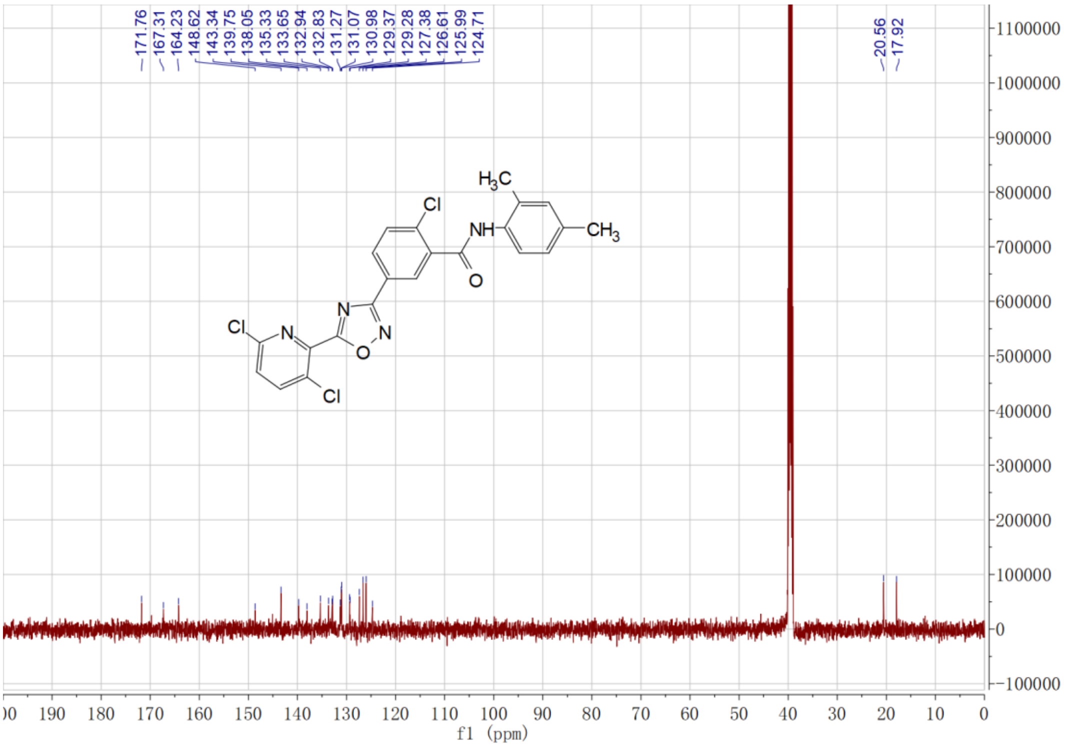

Figure S21 ^{13}C NMR spectra of **7g**

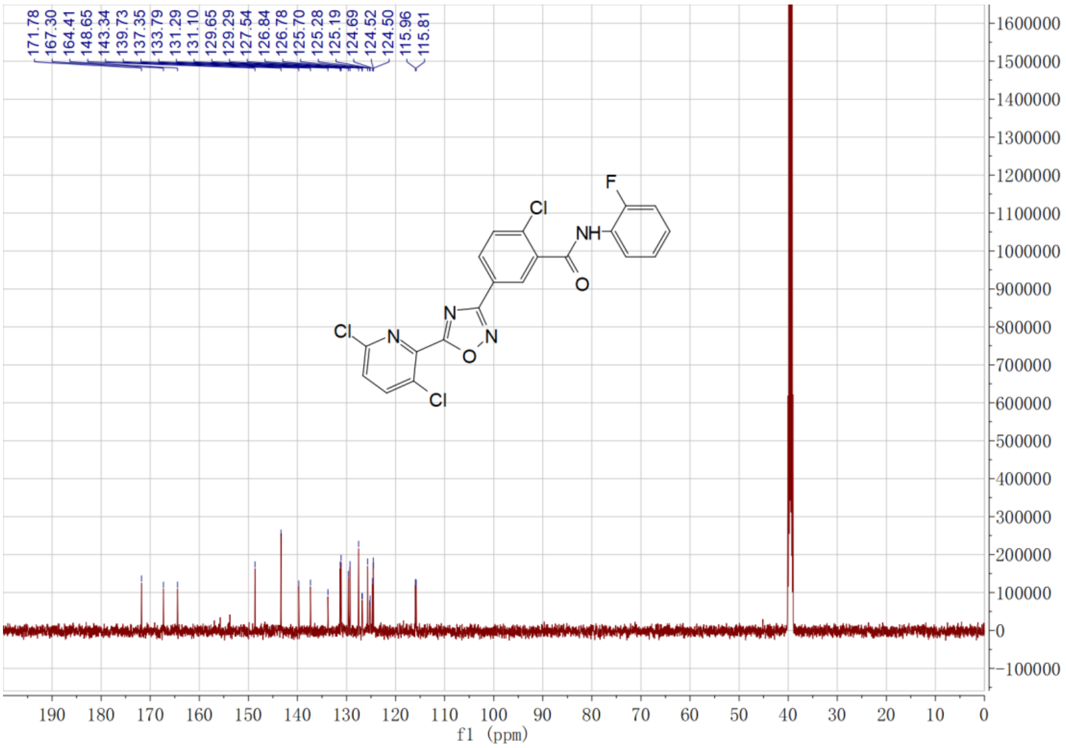

Figure S22 ^{13}C NMR spectra of **7h**

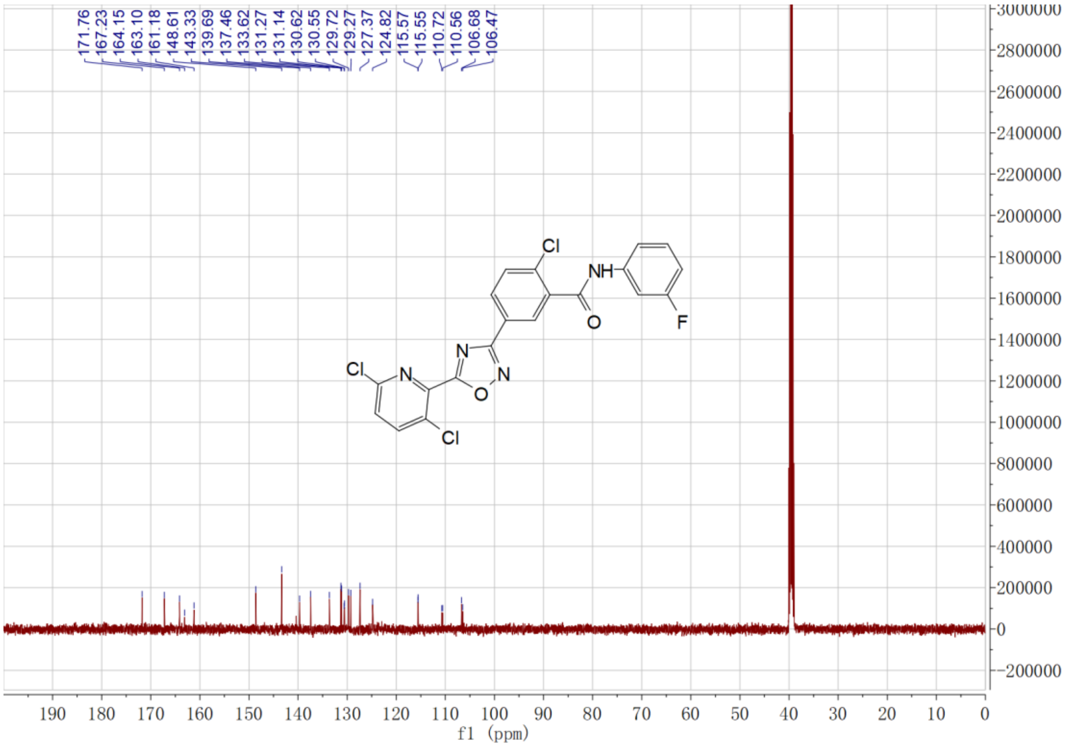

Figure S23 ^{13}C NMR spectra of **7i**

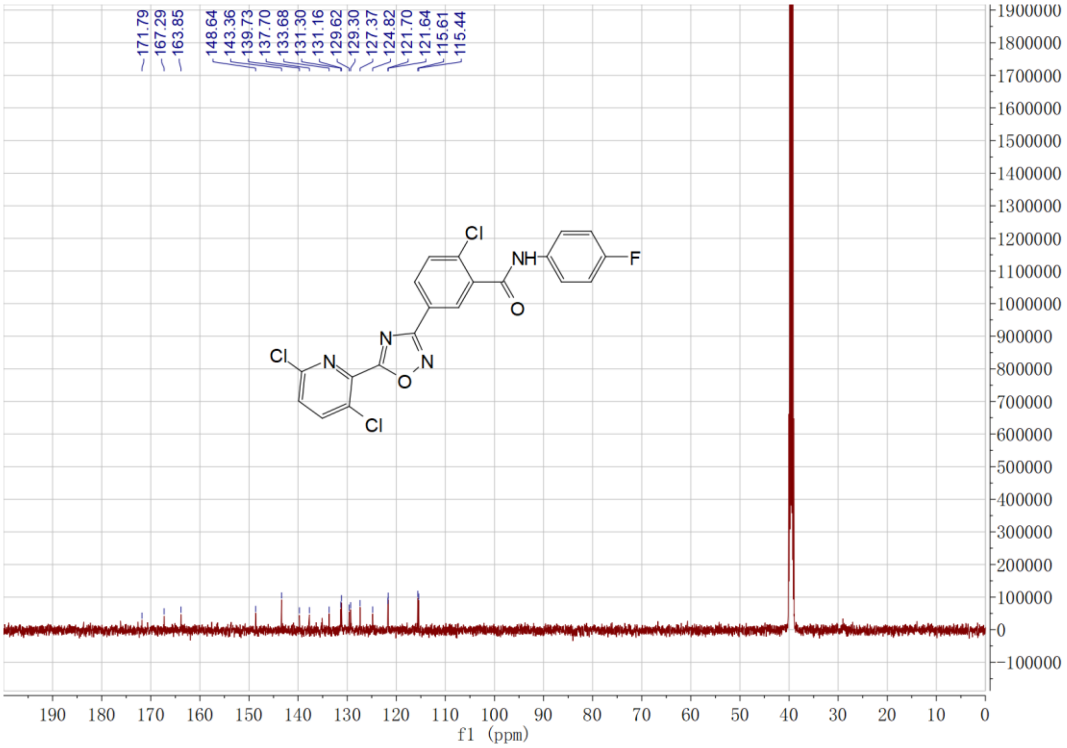

Figure S24 ^{13}C NMR spectra of **7j**

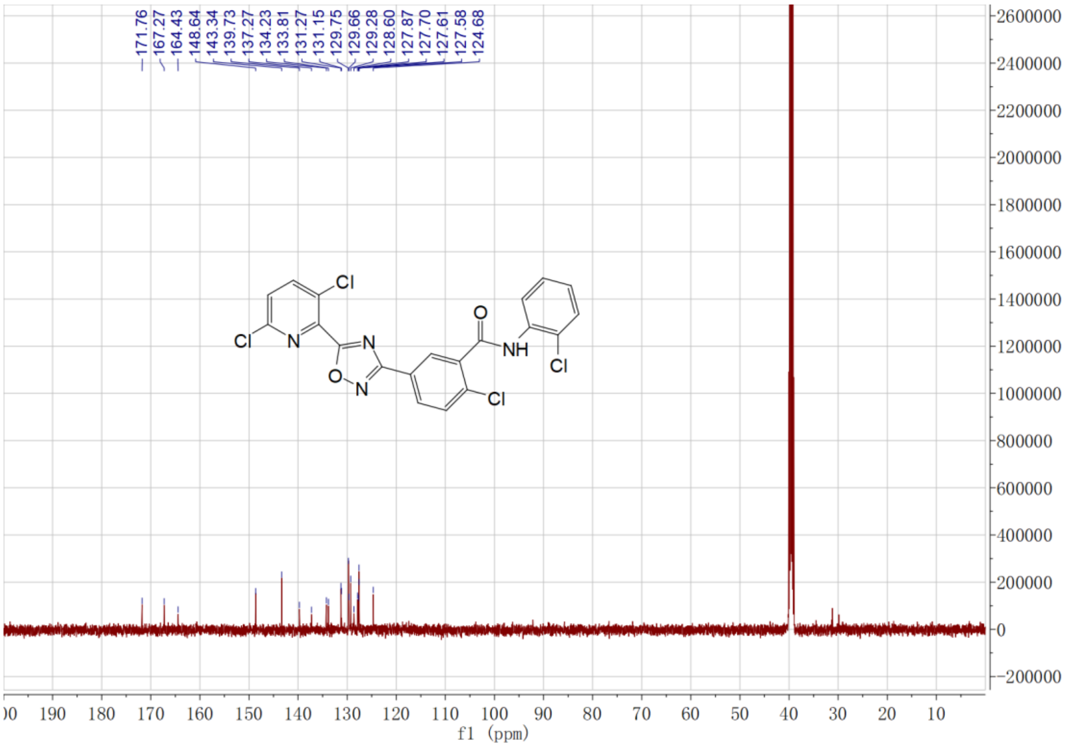

Figure S25 ^{13}C NMR spectra of **7k**

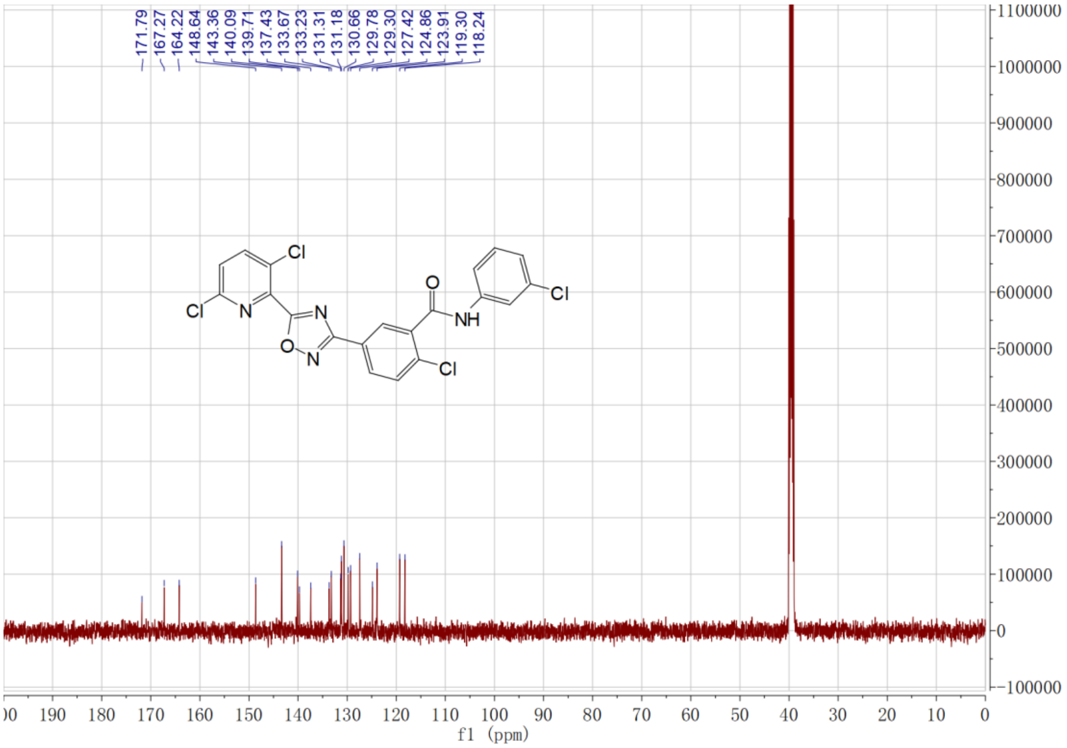

Figure S26 ^{13}C NMR spectra of **7l**

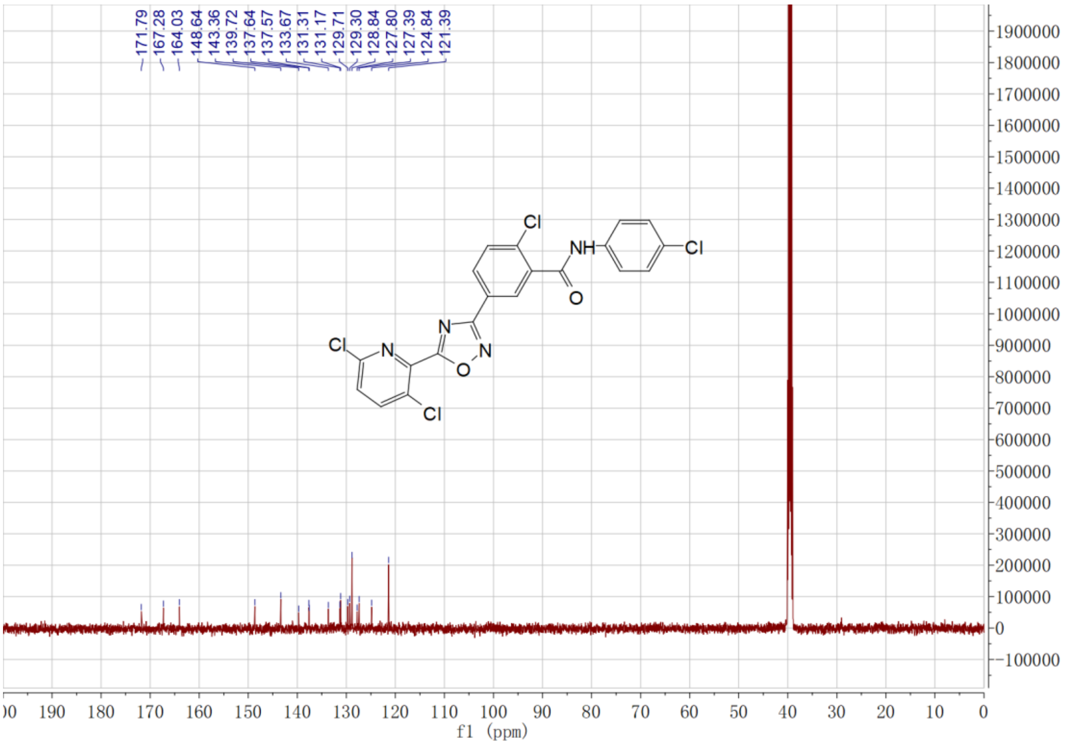

Figure S27 ^{13}C NMR spectra of **7m**

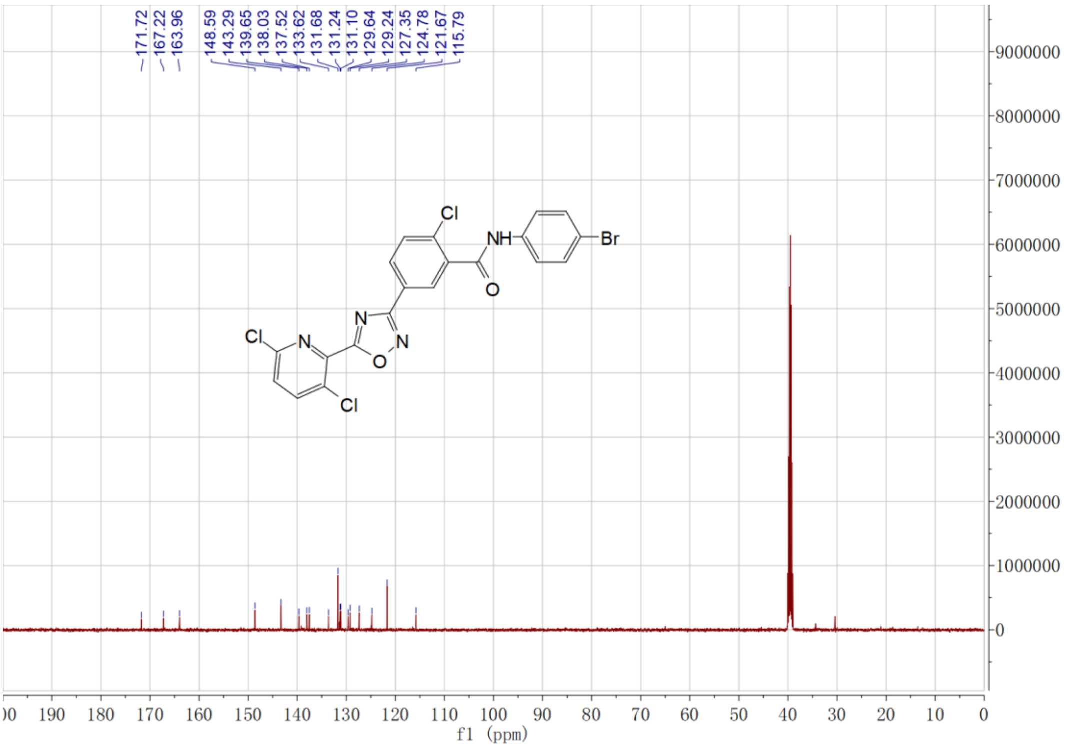

Figure S28 ^{13}C NMR spectra of **7n**

3. ESI-HRMS spectra of **7a~7n**

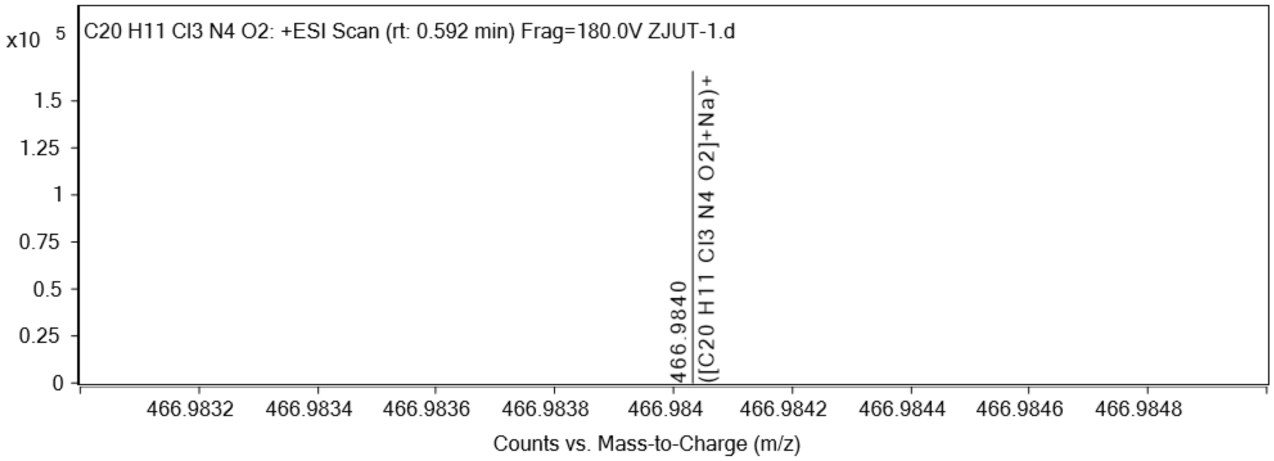

Figure S29 ESI-HRMS spectra of **7a**

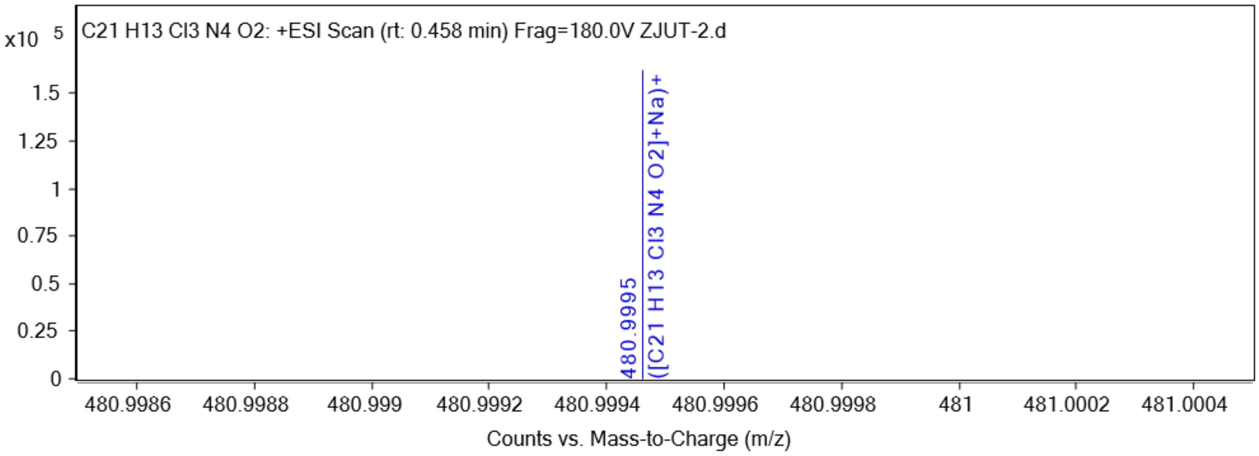

Figure S30 ESI-HRMS spectra of **7b**

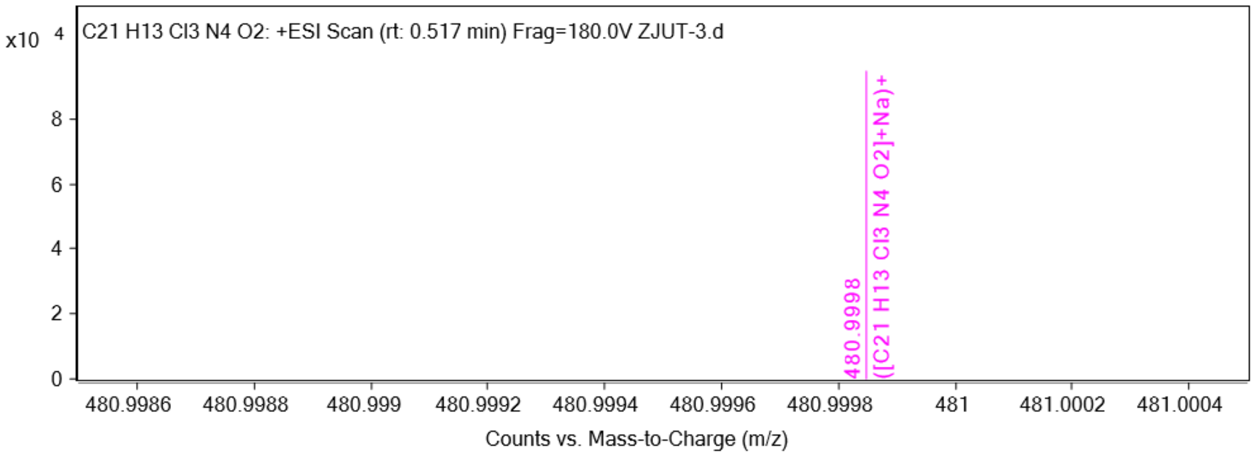

Figure S31 ESI-HRMS spectra of **7c**

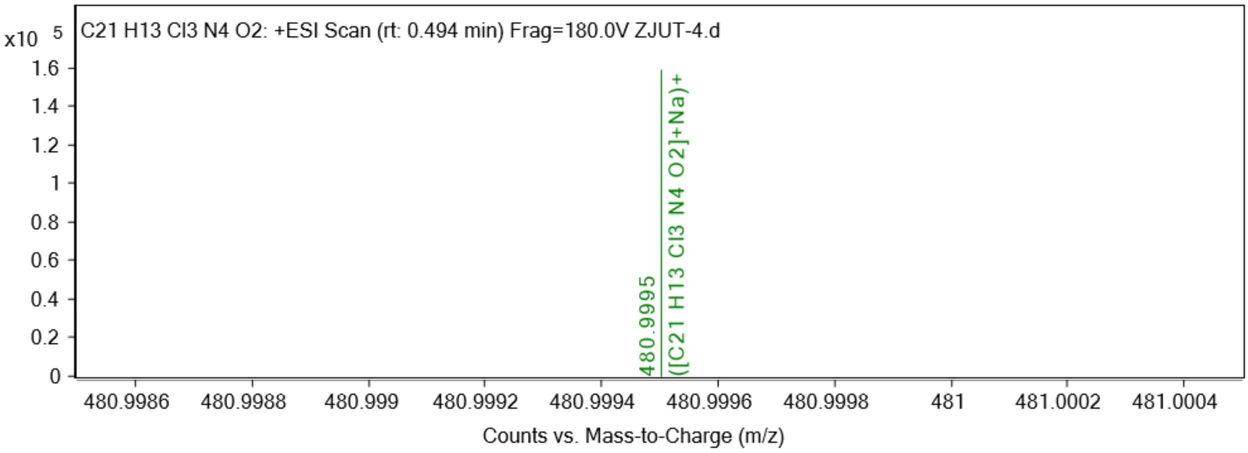

Figure S32 ESI-HRMS spectra of **7d**

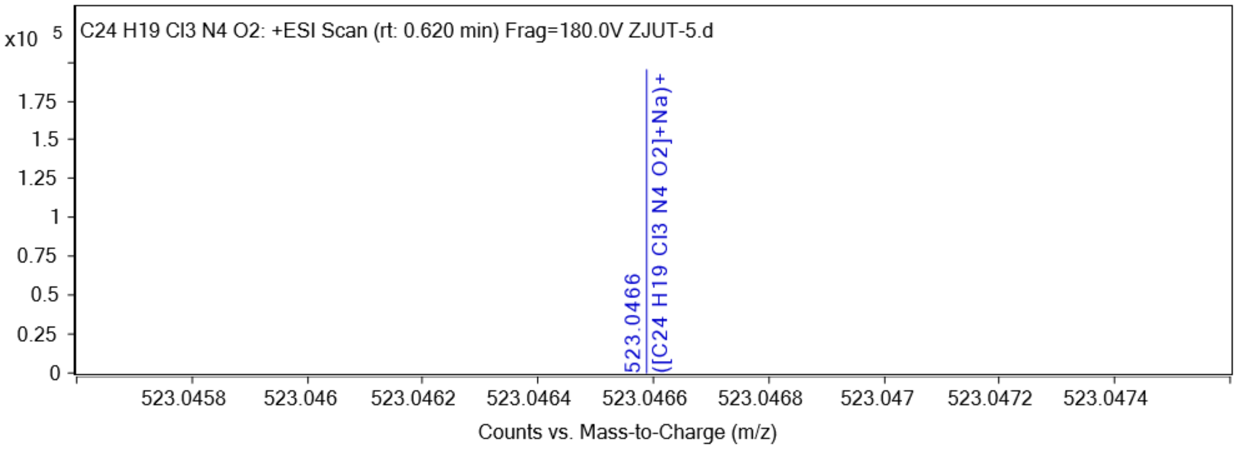

Figure S33 ESI-HRMS spectra of **7e**

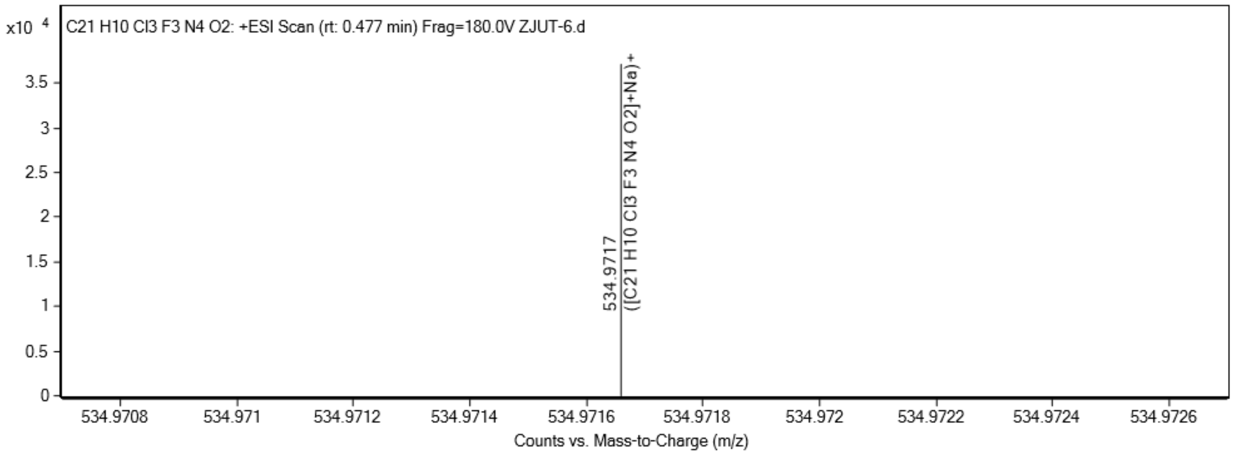

Figure S34 ESI-HRMS spectra of **7f**

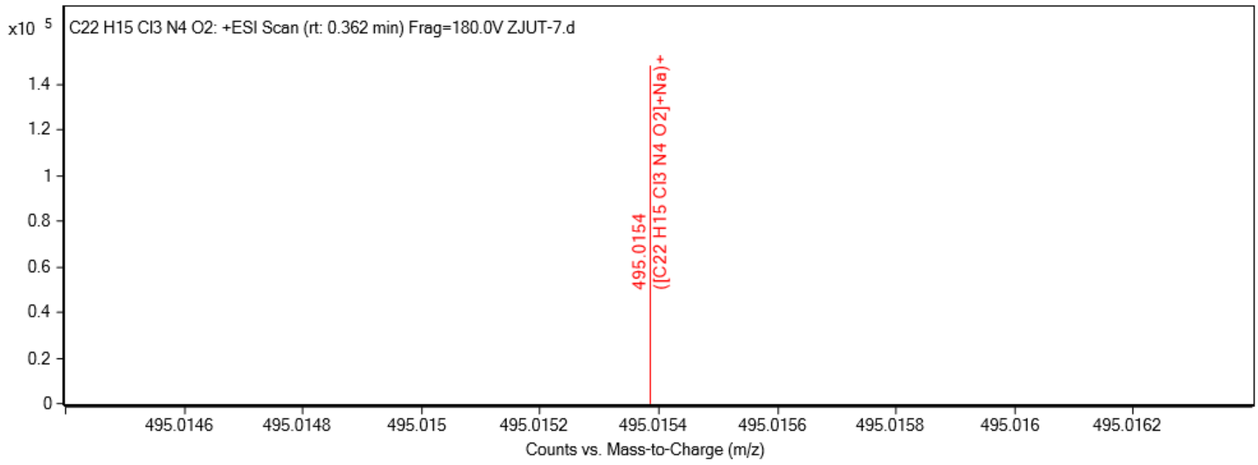

Figure S35 ESI-HRMS spectra of **7g**

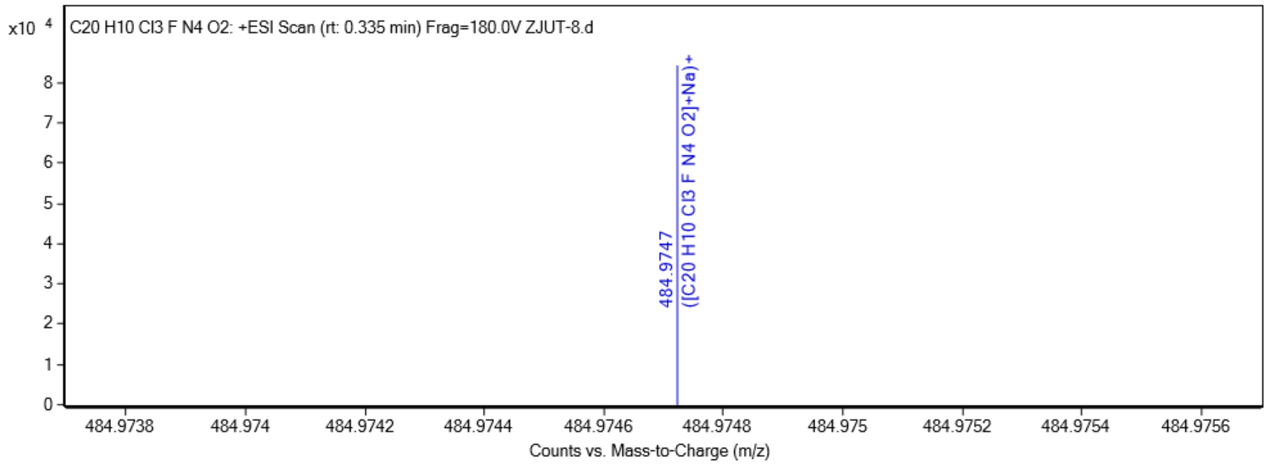

Figure S36 ESI-HRMS spectra of **7h**

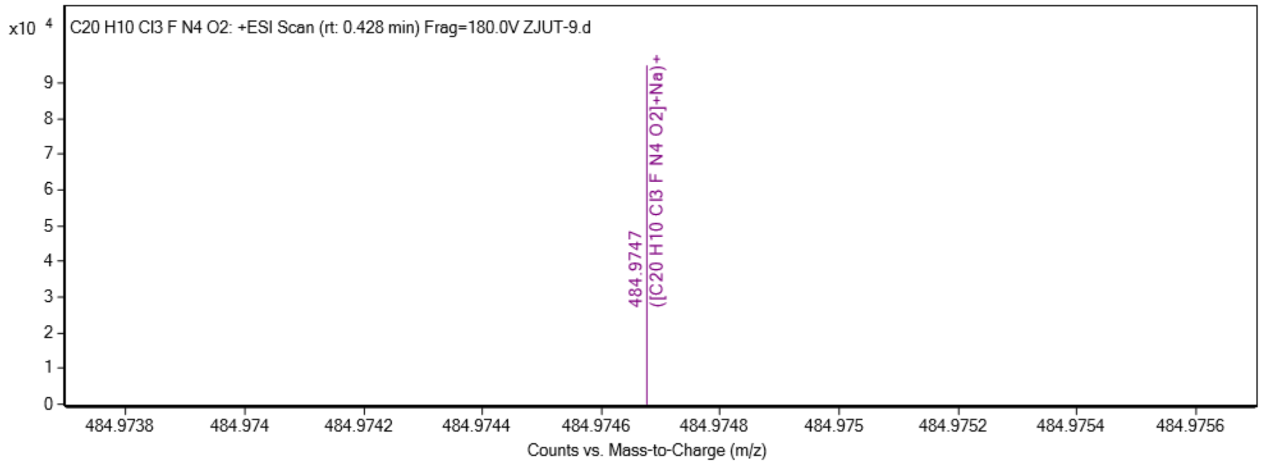

Figure S37 ESI-HRMS spectra of **7i**

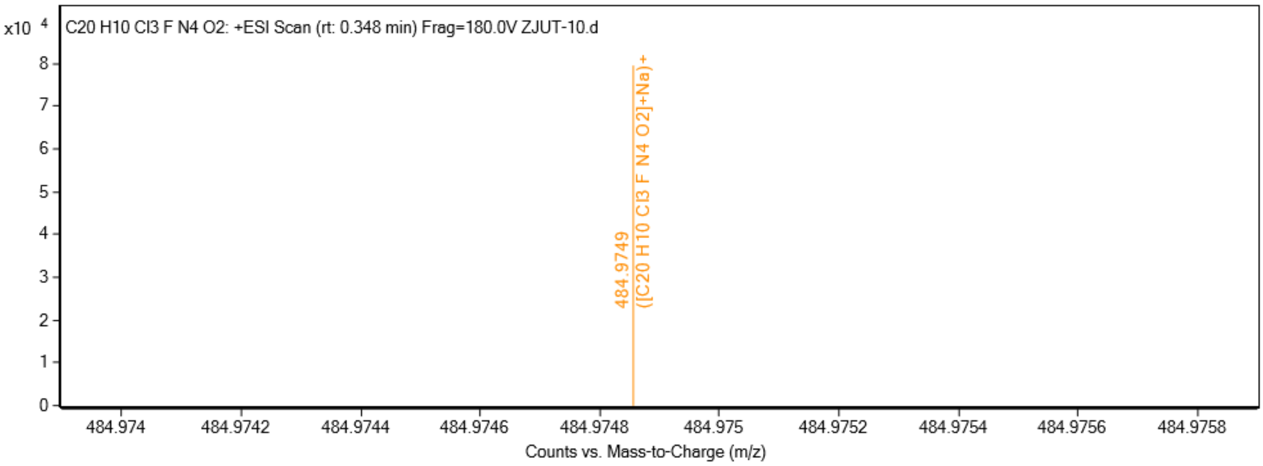

Figure S38 ESI-HRMS spectra of **7j**

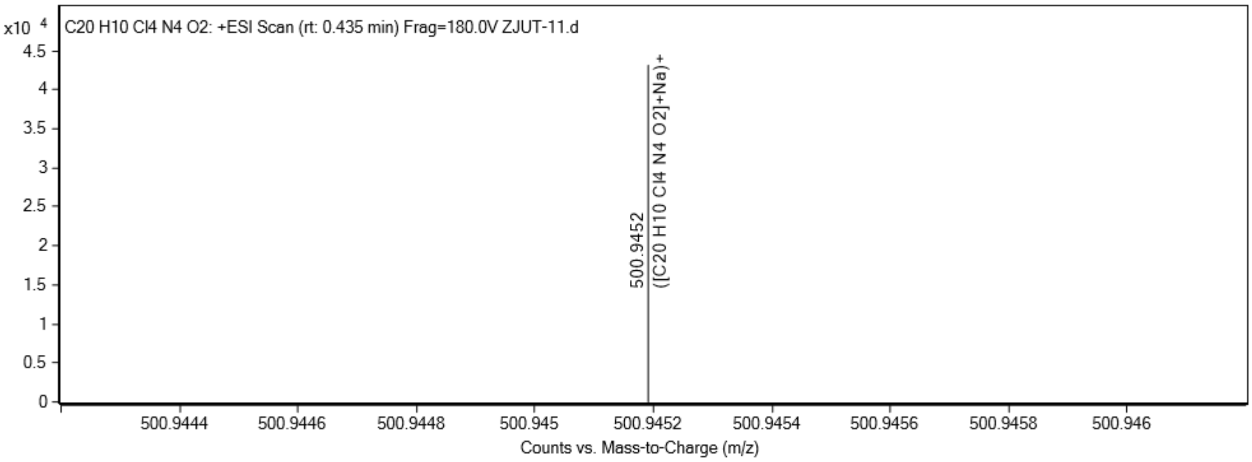

Figure S39 ESI-HRMS spectra of **7k**

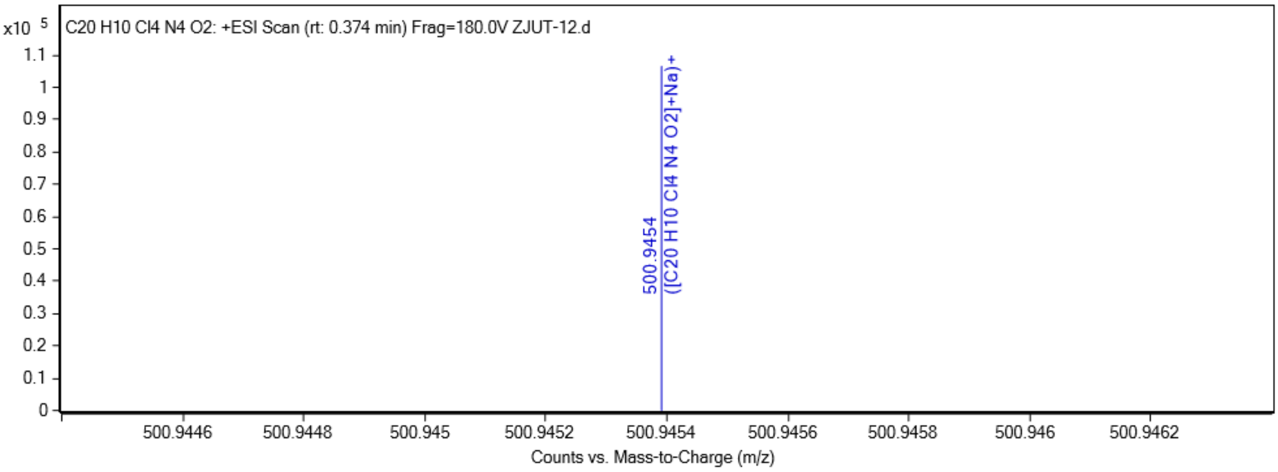

Figure S40 ESI-HRMS spectra of **7l**

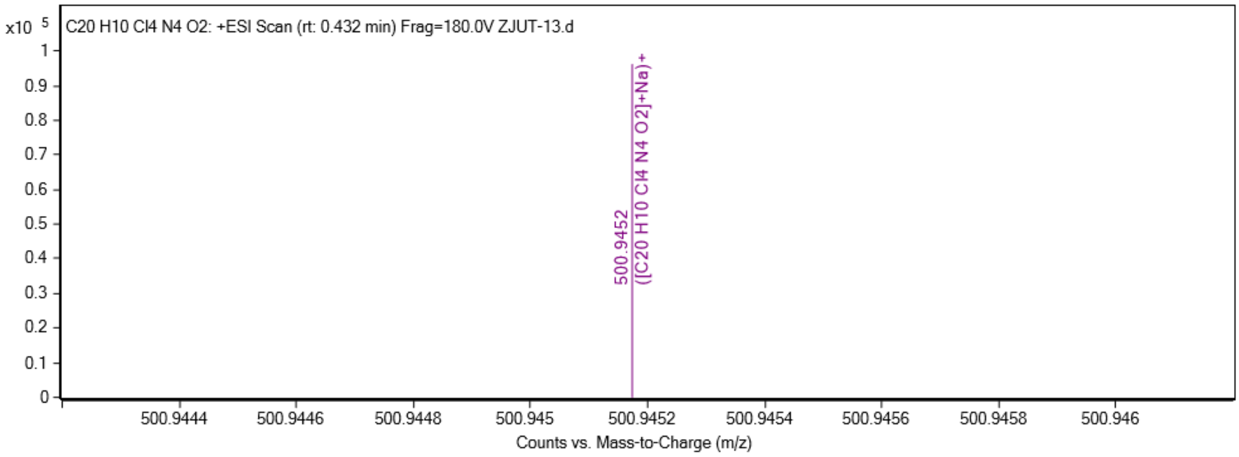

Figure S41 ESI-HRMS spectra of **7m**

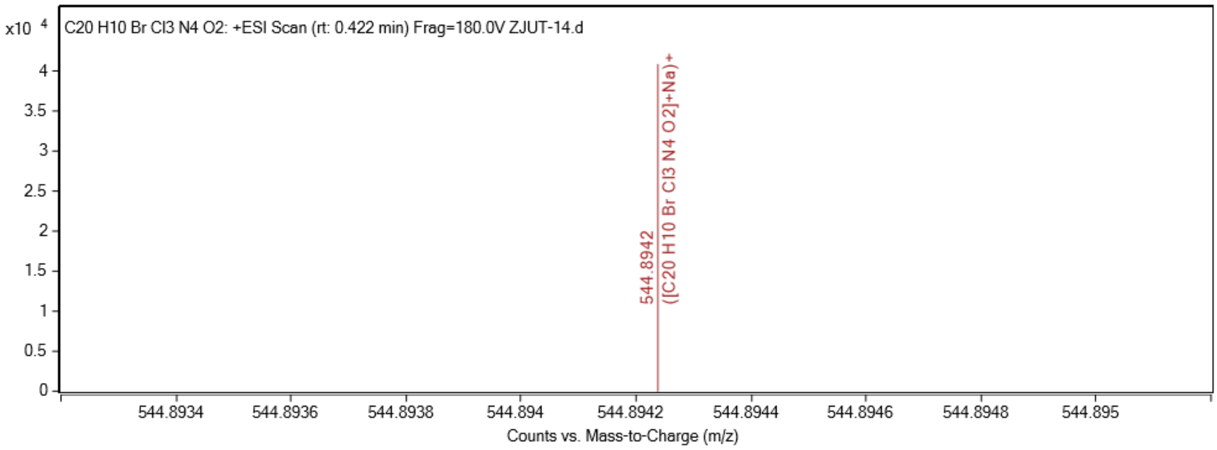

Figure S42 ESI-HRMS spectra of **7n**
